# Influence of comorbidity with depression on interdisciplinary therapy: outcomes in patients with chronic low back pain

**DOI:** 10.1186/ar3155

**Published:** 2010-10-11

**Authors:** Haili Wang, Carsten Ahrens, Winfried Rief, Marcus Schiltenwolf

**Affiliations:** 1 University of Heidelberg, Department of Orthopaedic Surgery, Schlierbacher Landstrasse 200a, 69118 Heidelberg, Germany; 2 University of Marburg, Department of Clinical Psychology and Psychotherapy, Gutenbergstrasse 18, 35032 Marburg, Germany

## Abstract

**Introduction:**

Our previous work showed higher tumour necrosis factor (TNF)-α levels in patients with chronic low back pain (cLBP) compared to healthy controls. However, patients with depression as a comorbidity did not have higher TNF-α levels in comparison to patients without depression. In this study we investigated the influence of depression on therapy outcomes such as TNF-α serum levels, pain intensity and back function in patients with cLBP. Our hypothesis was that patients with both cLBP and depression benefit no less than patients with cLBP alone from the multidisciplinary pain therapy.

**Methods:**

A total of 58 patients with cLBP alone or with both cLBP and depression were age- and sex-matched with 29 healthy controls. Serum concentrations of TNF-α were assayed at the beginning of the study (T0) and 10 days (T1), 21 days (T2), and 180 days (T3) later. The clinical outcomes such as pain intensity, as well as back function, sleep, exercise, alcohol and nicotine consumption were documented. In the first three weeks, all patients underwent multidisciplinary therapy based upon biological, psychological, physical and psychosocial components.

**Results:**

Over the whole course there were no differences in TNF-α level between cLBP patients with and without depression. At T0, both cLBP patients with (cLBP+DE) and without (cLBP) depression showed significantly higher TNF-α serum levels (*P *= 0.002 for cLBP+DE, *P *= 0.004 for cLBP) than healthy controls (HC) that normalized after 10 days of therapy and remained similar to healthy controls. During the follow-up, the depression scales were normalised and pain intensity was significantly reduced. Both evidences processed parallel to the reduction of TNF-α levels, which correlates neither with depression score nor with pain intensity at any time point.

**Conclusions:**

Depression as a comorbidity to chronic low back pain did not influence the serum TNF-α level in the course of six months, but seemed to affect the success of therapy. cLBP patients with comorbidity of depression benefit from multidisciplinary pain therapy not only to the same extent but also to a greater degree than cLBP patients without depression.

## Introduction

Patients with chronic pain have mostly additional psychiatric diseases. The range lists from the most to the fewest constellation include the affective diseases (depression), psychopharmaca associated diseases, somatoforme and anxiety disorders which are more frequent in chronic pain patients than in healthy subjects [1,2]. The Canadian National Population Health Survey found that the incidence of a major depression was doubled in patients with long-term back diseases [3]. Diseases with long-term persistent pain have higher risks of depression [4,5]. cLBP patients with depressive symptoms exhibit higher pain intensity, considerable impairment due to pain [6] and more passive avoidance behaviour than patients without comorbidity [7]. Depression is a risk factor for development and maintenance of chronic pain and long-term disturbance inclusive working place lost [8]. And depression seems to be a predictor for disability of chronic pain patients [9] and is associated with worse functional ability and more disorders due to pain [10]. The costs of medical treatment for cLBP patients with depression was found to be 2.8 times higher than those of patients without depression [11], and the economic cost of depressive disorders is higher in the presence of coexisting pain [12]. Summarized, depression and pain share the same neurotransmitter and pathways and influence each other, increase their prevalence and affect the treatment/treatment successes of one another [13]. Therefore, the accompanying depression should be recognized early and taken into account in the treatment strategy for chronic pain [14-17].

To date, however, pain and depression have been considered as separate entities and therefore treated in isolation. The number of therapy resistant patients with chronic pain who could not be helped by the conventional therapy and, therefore, still looking for further medical help, is calculated at about 400.000 to 700.000 in Germany. The reasons for the breakdown of conventional therapy are isolated therapy of assumed causal causes or mostly only drug therapy or passive treatment strategies [18] without consideration of psychopharmacological knowledge and the common ground of pain and depression.

Over the past 10 years we have performed in our clinic a multidisciplinary pain therapy which was based on biological, psychological, physical and psychosocial components. This therapy comprised six-hour sessions on five days per week for three weeks, giving a total of 90 hours [19]. The goal of this therapy is to restore the physical and psychosocial abilities of the patients, to expand their knowledge of back protection techniques and protective behaviour, to improve positive skills for individual coping and emotional controls, and to increase the patient's activity levels when they return to the workplace. It integrates physical exercises, ergonomic training, psychotherapy, patient education, behavioural therapy and workplace-based interventions in individual therapy and in group sessions.

Our previous work showed great successes with the multidisciplinary pain therapy [20,21], which also worked on reduction of elevated serum levels of TNF-α in patients with cLBP [22] and fibromyalgia[19].

We wonder whether the changes in TNF-α level could influence the development of depressive symptoms, if we follow up for six months the course of TNF-α level, depression scores parallel to pain intensity. The hypotheses of this prospective, longitudinal study were 1) patients with both cLBP and depression benefit to the same degree from the multidisciplinary pain therapy; 2) the changes of TNF-α level are associated with development of depressive symptoms.

## Materials and methods

### Subjects

All participants gave informed consent, and the study was approved by the local ethics committee of the University of Heidelberg, Germany. Participants were consecutively recruited from the Department of Orthopaedic Surgery of the University of Heidelberg. Each group of 29 patients with chronic low back pain (cLBP) alone or cLBP together with depression (cLBP+DE) were matched with 29 healthy controls (HC) by age and sex. This prospective longitudinal study was conducted over a period of six months (Table 1).

**Table 1:** Clinical and psychosocial data of all subjects

	cLBP+DE (n = 29)	cLBP (n = 29)	Mann-Whitney-U-Test
Sex: female/male (number)	17/12	17/12	*P *> 0.05
Age: mean (range) (years)	45.3 (20 to 69)	44.7 (24 to 68)	*P *> 0.05
Body mass index (BMI):	25.1 (18.9 to 37.4)	24.2 (17.7 to 33.3)	*P *> 0.05
Sleep (24 h)			
Day 0 = T0	5.25	6.08	*P *> 0.05
Day 21 = T2	5.4	5.62	*P *> 0.05
Day 180 = T3	5.95	6.11	*P *> 0.05
Exercise (24 h)			
Day 0 = T0	5.67 ± 5.09	5.28 ± 3.95	*P *> 0.05
Day 21 = T2	4.87 ± 5.28	6.27 ± 6.16	*P *> 0.05
Day 180 = T3	5.95 ± 5.60	5.18 ± 4.05	*P *> 0.05
pain 24 h: mean (VAS 0 to 10)			
Day 0 = T0	6.30 ± 1.89	5.13 ± 2.30	*P *> 0.05
Day 21 = T2	4.18 ± 2.04	3.75 ± 2.44	*P *> 0.05
Day 180 = T3	3.55 ± 2.63	4.44 ± 3.25	*P *> 0.05
pain 7d: mean (VAS 0 to 10)			
Day 0 = T0	6.29 ± 1.58	5.19 ± 2.27	*P *> 0.05
Day 21 = T2	4.61 ± 1.65	4.19 ± 2.50	*P *> 0.05
Day 180 = T3	3.90 ± 2.57	4.65 ± 2.86	*P *> 0.05
CES-D: (score)			
Day 0 = T0	32.81 ± 9.38	12.68 ± 6.64	*P *< 0.001*
Day 21 = T2	20.00 ± 11.20	9.00 ± 7.73	*P *< 0.001*
Day 180 = T3	19.50 ± 10.87	13.53 ± 10.74	*P *> 0.05
back function (R&M):			
Day 0 = T0	13.77 ± 5.26	10.19 ± 5.65	*P *= 0.022**
Day 21 = T2	9.48 ± 6.31	7.87 ± 5.08	*P *> 0.05
SF-36 PCS score			
Day 0 = T0	32.73 ± 7.97	31.24 ± 10.61	*P *> 0.05
Day 21 = T2	36.41 ± 7.76	35.93 ± 9.49	*P *> 0.05
Day 180 = T3	37.75 ± 7.80	41.37 ± 11.97	*P *> 0.05
SF-36 MCS score			
Day 0 = T0	28.95 ± 10.01	47.97 ± 6.26	*P *< 0.001*
Day 21 = T2	34.45 ± .66	52.08 ± 7.41	*P *< 0.001*
Day 180 = T3	45.77 ± 9,51	46.64 ± 11.24	*P *> 0.05
Alcohol consumption (24 h)			
Day 0 = T0	16%	19%	*P *> 0.05
Day 21 = T2	26%	8%	*P *> 0.05
Day 180 = T3	52%	19%	*P *= 0.03**
Nicotine consumption (24 h)			
Day 0 = T0	42%	23%	*P *> 0.05
Day 21 = T2	48%	29%	*P *> 0.05
Day 180 = T3	47%	17%	*P *= 0.041**

CES-D, German version of the Centre for Epidemiological Studies; cLBP, chronic unspecific low back pain; cLBP+DE, chronic unspecific low back pain and comorbidity of depression; MCS, Mental Composite Scale; pain 24 h, average pain in the last 24 hours; pain 7 d, average pain in the last seven days; PCS, physical Composite Scale; R&M, Roland & Morris Disability Questionnaire; SF-36, 36-item short form health survey; VAS, visual analogue scale. *: still significant after adjustment by Bonferroni-correction (< 0.005); **: not more significant after adjustment by Bonferroni-correction (> 0.005)

The inclusion criterion for pain was cLBP as the main symptom, defined as disabling pain of at least six months' duration that led to the patient being on sick leave for at least six weeks. Patients with other pain locations as their main symptom and patients with multiple major pain locations were excluded from this study.

The inclusion criteria for the diagnosis "depression" were: (i) an ICD-10 diagnosis of a current and an at least moderate depressive episode; (ii) a minimum CES-D score of 25.

Exclusion criteria in patients and controls were: tumour disease (diagnosis from history and by radiographic/MRI examination); trauma/fracture (history and radiographic examination); inflammatory systemic disease or infection, for example, spondylodiscitis (blood count and radiographic evaluation/MRI); nucleus pulposus prolapse with corresponding radicular pain (clinical examination, MRI); structural pathology of the lumbar spine, for example, spinal stenosis or spondylolisthesis (radiographic evaluation/MRI and clinical examination); rheumatological disease; serious cardiopulmonary, vascular or other internal medical conditions; any sensorimotor and/or neurological deficits in the lower extremity (clinical examination); spinal surgery in the year before admission to multidisciplinary therapy; radiographically apparent degenerative changes in the lumbar spine (grade II or above according to the Kellgren and Lawrence classification,1957 [23]; medication that may influence TNF-α level (for example, oral or local corticosteroids, aspirin, anti-TNF-α therapy); psychiatric disorder.

### Multidisciplinary pain therapy (MDPT)

Patients with cLBP or cLBP+DE underwent an in-patient course of multidisciplinary therapy with biological, psychological, physical and psychosocial components. This comprised six-hour sessions on five days per week for three weeks, giving a total of 90 hours [19]. The goal of this therapy is to restore the physical and psychosocial abilities of the patients, to expand their knowledge of back protection techniques and protective behaviour, to improve positive skills for individual coping and emotional controls, and to increase the patient's activity levels at their return to the workplace. It integrates physical exercises, ergonomic training, psychotherapy, patient education, behavioural therapy and workplace-based interventions in individual therapy and in group sessions.

After completion of this treatment program, the patients were discharged without further interventions by the hospital. They were allowed to contact the physician who had referred them for therapy, but they were advised to manage similar further pain episodes on their own without immediately contacting a physician. Further utilization of medical services after completion of the therapy program was not monitored.

### Evaluation

At study entry, the initial evaluation included clinical and radiographic examination and also MRI of the lumbar spine in all patients of the entire study, and blood count in all patients and controls. Each patient and control was evaluated at four fixed time points with investigations, including analysis of blood samples. The specific time points were the beginning of the study (T0), Day 10 (T1), Day 21 (T2) and finally at six months (T3) follow-up. Patients were additionally evaluated by standardized questionnaires and physical examinations at these time points.

#### Pain

Average pain intensity of all patients was determined from a visual analogue scale recording from 0 (no pain) to 10 (severe pain) during the last 24 hours and last week.

#### Back function

Measures of pain-related disability was assessed by using the Roland and Morris questionnaire [24], which is a self-administered questionnaire consisting of 24 item chosen to reflect varied activities of daily living. An item receives a score of 1 if it is checked as applicable by the respondent and a score of 0 if it is not marked. Accordingly, total scores can vary from 0 (no disability) to 24 (severe disability).

#### Depression

The CES-D is a well-established self-reporting instrument to assess the level of depression with 20 items and a potential overall score of 0 to 60. It has high specificity (94%) for the identification of acute depression if a score of at least 23 points is reached and the correlation coefficient to other instruments for measuring depression, such as the Hamilton Depression Scale, is acceptable (*r *= 0.49) and increases with recovery from depression (*r *= 0.86) [25].

To identify other confounding factors, at each time point the patients filled in this standardized questionnaire about sleep duration, alcohol and nicotine consumption, exercise and SF-36 questionnaire.

The SF-36 questionnaire was originally developed for the Rand Corporation's Health Insurance Experiment [26,27]. It is a self-administered, 36-item questionnaire that measures health-related functions in eight domains represented by eight scales. These generate two composite scales: PF = Physical Functioning, RP = Role Physical, BP = Bodily Pain, GH = General Health > PCS = Physical Composite Scale; VT = Vitality, SF = Social Functioning, RE = Role Emotional, MH = Mental Health > MCS = Mental Composite Scale. After summing the Likert-scaled items in the SF-36, each scale was then standardized so that the scores ranged from 0 (lowest level of functioning) to 100 (highest level).

To identify confounding factors of medication, drug intake in the two groups was studied accordingly to the ATC Classification System (ATC System).

### Determination of cytokine levels in serum

At the given time points, venous blood was taken from the cubital vein between 8 a.m. and 9 a.m. Blood samples were centrifuged at 2,000 rpm at 4°C within 30 minutes of withdrawal and serum was stored at -80°C. TNF-α serum levels were analysed in duplicate using a Bio-Plex cytokine assay (Bio-Rad Laboratories, Munich, Germany) according to the manufacturer's instructions. Median fluorescence intensity (MFI) of standards and patient samples were determined. Using the Bio-Plex Manager software, serum levels of TNF-α were deduced from the standard curve. The intra-assay coefficient of variation was 5 to 10%.

### Statistical analysis

In case of significant results, single *t-*tests with Bonferroni correction and a *P *< 0.05 was used. ANOVA tests were used for analysis of repeated measures. Correlations between the individual groups and cytokines were investigated using the χ^2 ^test, Fisher's exact test, and the phi coefficient. Drug intake was analysed as captured/non-captured without considering the dose. The nonparametric McNemar test was used to identify significant changes in medication during the six-month study period. Correlations of medication according to the ATC System with cytokine levels were calculated using the χ^2 ^test, Fisher's exact test, and the phi coefficient. *P-*values below 0.05 were considered statistically significant; those below 0.01, highly significant. The data were analysed using SPSS 15.0 software (SPSS, Chicago, IL, USA).

## Results

### TNF-α serum levels

**Figure 1:**
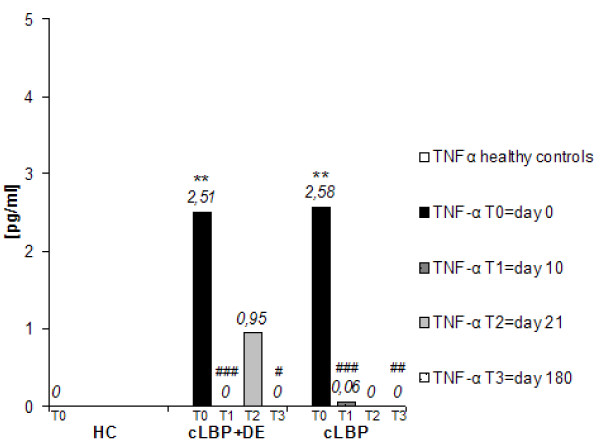
**Serum levels of pro-inflammatory cytokine TNF-α (pg/ml, median) in patients with cLBP and HC during 6 months' follow-up**. The serum level of TNF-α was measured using the Bio-Plex cytokine assay (Bio-Lab, Munich, Germany). The y-axis shows the median values of TNF-α serum levels (pg/ml). The Mann-Whitney-Test showed that serum levels of TNF-α of cLBP patients both with and without depression (DE) were significantly higher than those of healthy controls at day 0, but not at days 10, 21 and 180. **: *P *< 0.01, differences between patient groups and healthy controls; ###: *P *< 0.001, ##: *P *< 0.01, #: *P *< 0.05, decrease between T0 and T1 or T0 and T3. cLBP, chronic low back pain; HC, healthy controls.

At T0 both patient groups, with and without comorbid depression, showed significantly higher TNF-α serum levels than healthy controls (*P *= 0.002 for cLBP+DE, *P *= 0.004 for cLBP). At T1, T2 and T3, no differences in TNF-α serum level were found between patient group and the healthy controls (*P *= n.s.). No differences in TNF-α levels between the cLBP+DE group and the cLBP group were seen at any time (Figure 1).

The TNF-α levels in both back pain groups decreased significantly between T0 and T1 (both *P *< 0.001). From T1 to T2, only the cLBP+DE group showed a significant increase (*P *= 0.004) in TNF-α levels; in the cLBP group, TNF-α levels were unchanged. From T2 to T3, TNF-α levels in both cLBP groups decreased significantly (*P *= 0.023 for cLBP+DE, *P *= 0.038 for cLBP). At T3, TNF-α levels were significantly decreased in both cLBP groups compared to the level at T0 (*P *= 0.037 for LBP+DE, *P *= 0.009 for cLBP) (Figure 1).

### Depression scores

**Figure 2:**
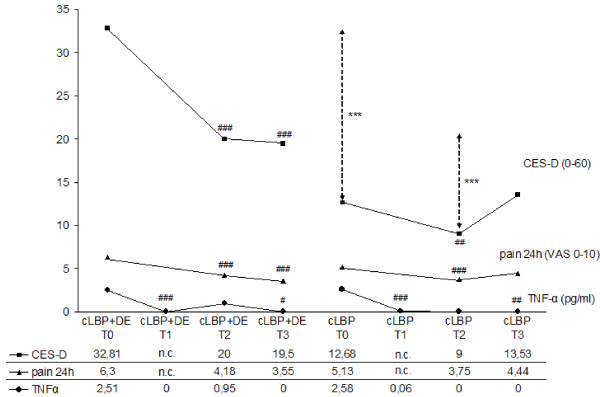
**Course of TNF-α serum level, pain intensity and depression score during six months' follow-up**. The two patient groups showed no differences in TNF-α serum level or pain intensity at any time point. Patients with depression showed significantly higher CES-D values than those without depression at t0 (*P *< 0.001) and t2 (*P *< 0.001), but not at t3 (*P *> 0.05). All three parameters were significantly lower at t2 and t3 than at t0, with the exception of CES-D at t3 in patients with isolated cLBP. n.c. = not collected. *: differences between patient groups (*** = *P *< 0.001); #: comparison of T1, T2 and T3 each vs T0 (### = *P *< 0.001, ## = *P *< 0.01; # = *P *< 0.05).

At all time points the CES-D scores were higher in the cLBP+DE group than in the cLBP group. There were significant differences between the two groups at T0 and T2 (*P *< 0.001 in both cases) but not at T3. At T3 (six months after therapy) the CES-D scores of the two groups were similar (Table 1). The mean CES-D scores of both groups decreased significantly from T0 to T2 (cLBP+DE: *P *< 0.001, cLBP: *P *= 0.009). Between T2 and T3 there were no significant changes. Over the total study period of six months only the comorbid cLBP+DE group showed a significant decrease (*P *< 0.001) (Table 2, Figure 2).

**Table 2:** Changes of TNF-α levels and clinical parameters during 6-months follow-up

		cLBP+DE (n = 29)	cLBP (n = 29)
TNF**-**α levels	T0/T1	↓***	↓***
	T0/T2	n.s.	n.s.
	T0/T3	↓*	↓**
CES-D scores	T0/T1	n.c.	n.c.
	T0/T2	↓*** 39%	↓** 31%
	T0/T3	↓*** 43%	n.s. (3% ↓)
Pain 24 h	T0/T1	n.c.	n.c.
	T0/T2	↓*** 36%	↓*** 26%
	T0/T3	↓*** 49%	n.s. (15% ↓)
Pain 7 d	T0/T1	n.c.	n.c.
	T0/T2	↓*** 31%	↓*** 41%
	T0/T3	↓* 17%	n.s. (11% ↓)
R&M scores	T0/T1	n.c.	n.c.
	T0/T2	↓*** 35%	↓** 25%
	T0/T3	n.c.	n.c.
Exercise 24 h	T0/T1	n.c.	n.c.
	T0/T2	n.s. (10% ↓)	n.s. (19% ↑)
	T0/T3	n.s. (17% ↑)	n.s. (13% ↑)
SF-36 PCS scale	T0/T1	n.c.	n.c.
	T0/T2	n.s. (8% ↑)	↑* 17%
	T0/T3	↑** 13%	↑* 25%
SF-36 MCS scale	T0/T1	n.c.	n.c.
	T0/T2	↑*** 25%	↑**11%
	T0/T3	↑** 57%	n.s. (4% ↑)

↑, increase; ↓, decrease; CES-D, German version of the Centre for Epidemiological Studies Depression Scale; cLBP, chronic unspecific low back pain; cLBP+DE, chronic unspecific low back pain and comorbidity of depression; MCS, mental Composite Scale; n.c., not collected; n.s., not significant; PCS, Physical Composite Scale; SF-36, 36-item short form health survey; T0/T1, difference between Day 0 and Day 10; T0/T2, difference between Day 0 and Day 21; T0/T3, difference between Day 0 and Day 180; TNF-α, tumour necrosis factor- alpha; VAS, visual analogue scale. *: *P *< 0.05; **: *P *< 0.01; ***: *P *< 0.001

### Pain intensity (last 24 hours and last week, numeric rating scale)

Regarding pain intensity of the last 24 hours and the last week both groups did not differ of each other at all time points. At T0 and T2 the comorbid cLBP+DE group showed a higher mean of pain whereas at T3 cLBP group displayed a greater intensity (Table 1). Patients of both groups reported significantly less pain at T2 than at T0 (pain 24 h: cLBP+DE and cLBP: *P *< 0.001, pain 7 d: cLBP+DE: *P *< 0.001, cLBP: *P *= 0.049). The cLBP+DE group still showed this significant difference in both pain intensities after six months at T3 (*P *< 0.001), at which time they demonstrated a 49% (24 h) and 41% (7 d) amelioration of perceived pain compared to T0, while patients with only cLBP improved their perceived pain only 15% (24 h) and 11% (7 d) in the total study period (each n.s.) (Table 2, Figure 2).

### Back function (Roland and Morris Disability Questionnaire)

Patients with both cLBP and depression (cLBP+DE) showed worse back function than patients with cLBP alone at T0 (*P *= 0.022), but not at T2. Three weeks after initiation of the therapy both groups of patients displayed improvements in back function: 35% in cLBP+DE and 25% in cLBP. These changes were significant in both groups (*P *< 0.001 for cLBP+DE, *P *= 0.001 for cLBP) (Table 1).

### Habits (sleep, exercise, alcohol and nicotine consumption)

There were no differences in reported sleep duration (in the past 24 hours) or alcohol and nicotine consumption (in the past 24 hours) between cLBP+DE and cLBP alone at the beginning of the study period, or between admission and discharge in cLBP+DE. However, at the end of the study a higher proportion of the formerly depressive cLBP patients reported consumption of alcohol (cLBP+DE 52%, cLBP 19%; *P *= 0.037) and nicotine (cLBP+DE 48%, cLBP 17%; *P *= 0.041) in the previous 24 hours (Table 2). The two patient groups did not differ in exercise level of the previous 24 hours at any time point (Table 1).

### SF-36 physical Composite Scale (PCS) and Mental Composite Scale (MCS)

The PCS scores of both patient groups did not differ at any time points. Compared to T0, cLBP patients displayed an amelioration of 13% (*P *= 0.006) at T2 and of 25% at T3 (*P *= 0.01), while cLBP+DE patients improved the PCS for 8% at T2 (*P *> 0.05) and 17% at T3 (*P *= 0.036) respectively.

The MCS scores was significantly higher in cLBP group than cLBP+DE at T0 (*P *< 0.001) and T2 (*P *< 0.001) but not at T3. Compared to T0, cLBP+DE patients improved the MCS for 25% at T2 (*P *< 0.001) and 57% at T3 (*P *= 0.002) respectively, while cLBP patients displayed an amelioration of 11% at T2 (*P *= 0.001) and of 4% at T3 (*P *> 0.05).

### Medication

#### Daily intake of medication

There was no significant difference in medication (inclusive antidepressants) intake between the two groups at any time point, except that patients in the cLBP+DE group were taking significantly more NSAIDs (M01A) than patients with cLBP alone at T0 (*P *= 0.037).

#### Intake of medication in the past 24 hours

No significant differences were found between the two groups. Comparing T0 and T3, significant reductions in daily intake of analgesics from 28% to 3% (N02, *P *= 0.039) and of NSAIDs from 28% to 7% (M01A, *P *= 0.031) were observed in the cLBP+DE group and of opioids (N02A, *P *= 0.031) from 28% to 7% in the cLBP group.

### Correlation between TNF-α serum level and confounding factors

No correlation was found between TNF-α serum level and age, sex, BMI, CES-D score or back function. The single correlation between TNF-α serum level and pain in the previous week was found at T3 (*P *= 0.046; phi coefficient = 0.490) in the comorbid depression group (cLBP+DE), but not in the cLBP group without depression. A negative correlation between TNF-α serum level and intake of analgesics was found at T0 in cLBP+DE (*P *= 0.027, S = --0.411) and a positive correlation between TNF-α serum level and NSAIDs at T3 in cLBP (*P *= 0.022, S = .486). But all these significances disappeared after adjustment by Bonferroni-correction.

## Discussion

To our knowledge, this is the first study which investigates the co-existence of pain and depression regarding circulating TNF-α in a longitudinal design. As we know, the pro-inflammatory cytokine TNF-α plays a role both in pain and depression. Both patients with depressive symptoms and patients with pain disorders often display enhanced cytokine levels. A recent review pointed out that the pathophysiologies of pain and depression may overlap in many respects [28]. It has been demonstrated that several brain regions are implicated in both major depressive disorder (MDD) and pain. For example, the insular cortex [29,30], the prefrontal cortex [31,32], the anterior cingulate cortex [33,34], the amygdala and the hippocampus [35-37] are activated and/or altered in response to both depression and pain. Moreover, Robinson [28] verified that shared neurocircuits and neurochemicals play an important role connecting the pathophysiologies of depression and pain disorders. However, the mechanisms of the interaction between cytokine, pain and depression are so far not clarified. Few studies address this focus, up to now.

In the current study, we tried to find out some evidence for the connection between TNF-α, pain and depressive symptoms. From the current study we learned that patients with chronic low back pain displayed a chronic systemic inflammation, independent from the presence of depression or not. Patients with both cLBP and depressive symptoms had worse back function and mental composite scale than those without depression at the beginning but not at the end of the study (Table 1). Otherwise, they did not differ from each other during the whole course, regarding TNF-α level, exercise activity, physical composite scale, sleep duration and pain intensity (Table 1).

**Figure 3:**
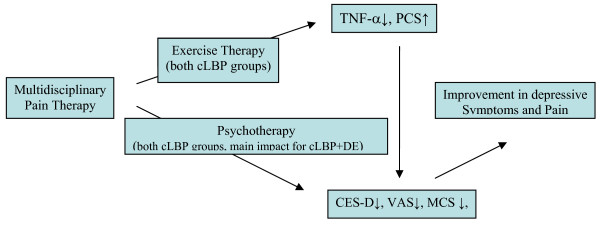
**The explanation of the current findings**. CES-D, German version of the Centre for Epidemiological Studies Depression Scale; cLBP, chronic low back pain; DE, depression; MCS, mental Composite Scale; PCS, physical Composite Scale; TNF-α: tumour-necrosis-factor α; VAS, visual analogue scale.

Besides, we found from this study that MDPT somehow managed the decline of circulation TNF-α first, and subsequently the pain and depression impairment. Patients with both cLBP and depressive symptoms improved more in their clinical parameters regarding CES-D scores, pain intensity, R&M scores, MCS scale than those without depression (Table 2) who just gained a greater reduction in TNF-α level and PCS scale. The outcome of this is the following explanation of our findings (Figure 3):

Supposedly due to exercise therapy within MDPT the circulating level of TNF-α decreased and the following improvement in clinical parameters three weeks after is a result of TNF-α reduction.

Due to intensive psychotherapy or synergetic effects of exercise and psychotherapy, the depression scores decreased and subjective pain perception, back function, mental composite scale were improved, while TNF-α serum level returned to a level without distinction to healthy controls.

Six months after the therapy, we noticed that parameters related to psychological elements such as depressive scores, mental composite scales, physical composite scales and even subjective visual analogue scores of pain stayed constantly and strongly improved in both patient groups from the beginning of the therapy. The interesting and, maybe also groundbreaking, fact from this study was that patients with both pain and depression showed stronger improvement in psychological elements such as depression scores, pain intensity, back function and mental composite scales while patients with only cLBP showed more improvement in physical elements such as physical composite scales. The course of TNF-α serum level was similar in both groups but the decrease over the total study period was higher in patients with only cLBP. This may point out that the pain in cLBP patients with depression is attributable to somatic symptoms of depression rather than primary pain, because patients with a comorbidity of depression respond more favourably to an intensive intervention involving significant interpersonal contact and support as they did during psychological elements of MDPT. That is why the improvement in pain was greater in the comorbid cLBP+DE group.

In the current study, the reduction of elevated TNF-α serum levels in both cLBP patient groups after MDPT may be considered as a psychoneuroimmunological correlate to the physical therapy and psychotherapy within the framework of MDPT. We guessed that one factor of MDPT (physical exercises, ergonomic training, psychotherapy, patient education, behavioural therapy and workplace-based interventions in individual therapy and in group sessions) or more factors together play a role in the normalization of TNF-α serum level so that the synergistic effect of all elements trigger the reduction of depressive and pain scores. The mechanisms can be explained as follows:

In regard to exercise therapy, it is known that exercise modulates the inflammatory response through neuroendocrine mechanisms. All exercise-induced changes in the innate/inflammatory response are mediated by stress hormones, mainly gluococorticoids and catecholamines [38,39]. Since the cLBP patients displayed chronic inflammation and seem to be associated with a deregulation of the inflammatory response state [22,40], which could cause an unbalanced hypothalamic-pituitary-adrenal axis and sympathetic nervous system activity, it could be plausible that exercise therapies could work at this level in cLBP patients.

In terms of psychotherapy, to date, randomized controlled trials have supported the efficacy of cognitive behavioural treatment (CBT) in treating both major depression [41] and pain disorders [42,43]. For the treatment of major depressive disorders (MDD), the combination of pharmacotherapy with CBT is optimal in some patient populations, such as chronic depression [44] and chronic pain conditions [42,45]. A meta-analysis of psychotherapeutic approaches for chronic lower back pain showed improvements of pain and depression in response to CBT and self-regulatory treatment [46]. It seems that the multidisciplinary nature of the pain therapy may have an especially persistent positive impact on health-related quality of life in the presence of manifest depression. In the present study, the significant difference between the two back pain groups in the mental composite scale (MCS) can be explained by the significant moderator influence of the variable of depressivity. The mean value for MCS was significantly lower in the group cLBP+DE than in the group cLBP at the first two time points (Table 1). The MCS value of the comorbid back pain group was lower than in the German reference sample at all time points, whereas that of the cLBP group was never significantly below that of the German reference group. After six months the MCS value in the cLBP+DE group was almost identical with that of the cLBP group, only slightly below it, which also speaks for the long-term effect of multimodal therapy with regard to improvement in mental health.

Potential confounding factors for TNF-α serum levels were analysed in this study. For many clinical parameters such as age, sex, BMI, sleep duration, exercise activity, pain intensity, physical composite scores were comparable between patients of both groups. Age, sex, BMI, CES-D score and back function did not correlate with TNF-α serum level. The single correlation between TNF-α serum level and pain during the previous week was found at T3 in the comorbid group but not in the cLBP group without depression. The fact that at the end of the study a higher proportion of the formerly depressive cLBP patients reported alcohol and nicotine consumption in the previous 24 hours (Table 2) seems not to be relevant for TNF-α level, pain intensity and severity of depression, because none of these parameters differed between patients with and without depression at T3 after adjustment by Bonferroni-correction.

The influence of medication on TNF-α serum level was also studied. It is known that tricyclic antidepressants could lower the production of pro-inflammatory cytokines like TNF-α, IL-1ß and IFN-r [47-49]. And improvement of symptoms regarding depression by cytokines was caused by the inhibition of production of pro-inflammatory cytokines and elevation of anti-inflammatory cytokines [50]. NSAIDs may inhibit the enzyme COX-1 and COX-2, and therefore, reduce the serum level of IL-6, NO and cortisone [51,52]. Even opioids influence the cytokine production and shift to a increasing of anti-inflammatory reaction [53,54]. In the current study, there was no difference in the use of antidepressants and opioids between patients with and without depressive symptoms. We found a negative correlation between TNF-α serum level and analgesic intake at the beginning of the study in the cLBP+DE group and a positive correlation between TNF-α serum level and NSAIDs at the end of the study in the cLBP group.

A possible explanation for the decline of TNF-α serum levels is that patients with comorbid depression took significantly more NSAIDs at T0 (28%) than patients with cLBP alone (7%) and used these drugs significantly less at T3 (7%) than at T0. NSAIDs are able to inhibit TNF-α production and TNF-α serum level at T0 was similarly high in both groups although NSAIDs intake in comorbid group was significantly greater at T0. So it is possible that the TNF-α serum level was higher in the comorbid group and reduced by NSAIDs intake at T0. But there is no correlation between NSAIDs and TNF-α in cLBP+DE group. In the course of the study TNF-α serum levels of boths groups were significantly lowered from T0 to T3 without a distinction to the level healthy controls at T3. In summary regarding TNF-α we suggest that multidisciplinary pain therapy may be responsible for the normalisation of its level without an impact of medication.

One primary limitation of our study is the small sample size. Therefore, the results from this study should be further confirmed in a larger collective. However, our study addresses for the first time a timely topic regarding the shared biological underpinnings of pain and depression, and it employs longitudinal methodology. Our results indicate therapeutic options that target both pain and depressive disorders including cognitive behavioral therapy, exercise, and pharmacotherapy. Future research is needed to further clarify the multiple interactions of pain and depressive disorders, including effects on neurohormonal-cytokine interaction, which will aid in the development of more effective treatment strategies addressing all symptoms with which a patient might present.

## Conclusions

The findings of this study suggest that depression as a comorbidity to cLBP did not influence the serum TNF-α level during six months' follow-up, but did affect the success of therapy. cLBP patients with comorbid depression benefited more strongly from multidisciplinary pain therapy than cLBP patients without depression. We assume that one or more elements of the therapy (whether psychotherapy, physical therapy, physiotherapy or others) may improve subjective pain perception, mood and immune function (in this case, TNF-α serum level). However, this needs to be confirmed in a further study involving a larger collective.

## Abbreviations

ATC: Anatomical Therapeutic Chemical Classification; CBT: cognitive behavioural treatment; CES-D: German version of Centre for Epidemiological Studies Depression Scale; ICD-10: International Statistical Classification of Diseases and Related Health Problems: 10^th ^Revision; IL: interleukin; MCS: Mental Composite Scale; MDD: major depressive disorder; MDPT: multidisciplinary pain therapy; NSAIDs: non-steroidal anti-inflammatory drugs; PCS: Physical Composite Scale; SF-36: 36-item short form health survey; TNF-α: tumour necrosis factor alpha.

## Competing interests

The authors declare that they have no competing interests.

## Authors' contributions

HW conceived the hypothesis for the manuscript, participated in data collection, wrote the first draft of the manuscript and had primary responsibility for the manuscript process. CA participated in data collection, performed the statistical analyses, contributed to and approved the final manuscript. WR participated in the interpretation of data, and contributed to and approved the final manuscript. MS conceived the study and participated in its design and helped to draft the manuscript. All authors read and approved the final manuscript.
